# Clinical Lecture—Asthma, Vesicular Emphysema, Hay Fever

**Published:** 1881-12-20

**Authors:** Robert C. Word

**Affiliations:** Professor of Physiology and Lecturer on Hygiene in the Southern Medical College, Atlanta, Georgia


### Southern Medical Record:

EDITORS:

T. S. POWELL, M.D. W. T. GOLDSMITH, M.D. R. C. WORD, M.D.

*R. C. WORD, M.D., Managing Editor.*

☞All Communications and Letters on Business connected with the RECORD must be addressed to the Managing Editor.

VOL. XI. ATLANTA, GA., DECEMBER 20, 1881. No. 12.

### ORIGINAL AND SELECTED ARTICLES.

### CLINICAL LECTURE.

### ASTHMA—VESICULAR EMPHYSEMA—HAY FEVER.

BY ROBERT C. WORD, M. D.,

Professor of Physiology and Lecturer on Hygiene in The Southern Medical College, Atlanta, Georgia.

GENTLEMEN—I propose to occupy the present hour with some remarks upon the subject of Asthma, and certain affections of a kindred nature which not unfrequently present themselves in practice.

We have before us a somewhat rare instance of recovery from what is called *Vesicular Emphysema* in a little girl 7 years old. The case was regarded and treated as asthma until she fell into my hands more than 12 months ago, and under treatment then adopted, which I will presently give you, she has almost wholly recovered. Her condition for a long while was one of much suffering from dyspnœa occasioned by dilatation of the air cells, and brought about by a severe and prolonged attack of whooping cough. The case was complicated with an engorged liver and some dropsical effusion, palpitation of the heart, cough, etc.

For several months she has been free from all the distressing symptoms, and no marks of the disease are now perceptible, save a slight tendency to cough at times, and the protuberant eye-balls, a condition often observed in asthmatics. The same symptom in greater degree, and accompanied by tumefaction of the thyroid gland and more or less heart lesion, is seen in what has been styled *exophthalmic goitre.*

*Asthma.*—Asthma may be defined a paroxysmal contraction of the circular muscular fibres of the minute bronchial tubes.

The word asthma is from the Greek *Asthmadzo*, to gasp for breath. The contraction of the circular fibres of the bronchi does not, as we believe, constitute the disease, but is an effect of an influence from without either direct or reflex; usually, we think, the latter. The irritating impression may proceed from the gastric or pulmonary branches of the pneumogastric nerve, or may have its remote seat in the medulla oblongata.

*Symptoms.*—Asthma usually makes its onset at night, and often suddenly; the patient is seized with oppression in the chest, difficulty of breathing, and a short, tight cough. These symptoms increase, and sometimes become exceedingly laborious and distressing with every appearance of suffocation. The patient rises from bed, calls for fresh air; his countenance becomes more or less anxious, his heart palpitates violently, his respiration is harsh, tight and wheezing, his lips are purple, and he is unable to lie down but sits erect, or leans forward upon a chair or table, to facilitate the action of the respiratory muscles.

If the ear be applied to the chest, tumultuous action of the heart will be heard with loud wheezings, sibilant rouchi and whistling sounds. The eyes, during the paroxysm, have a staring expression. The clinical thermometer will commonly show an abnormal fall of temperature during the more violent stage of the attack, reaching sometimes to 84° or lower.

The violence of the paroxysm usually subsides as day approaches, and during the day the patient is comparatively better; but it is apt to recur at night, and frequently continues to recur in exascerbations at night and remissions at day for a number of days.

It is not common in young people, and when it does occur in a child it is usually dependent upon hereditary predisposition.

Persons subject to asthma are more liable to attacks during cold, damp weather, especially during the prevalence of east winds; the atmosphere of certain localities, and the dust of ipecac and various other substances are liable to bring on an attack. I will not dwell upon the symptoms which you will find fully detailed in the text-books. Nor need I say much upon its pathology, which, after all that has been written upon it, yet remains an unsettled question. I cannot say that any material advancement has been made upon the pathological views of some of the older writers.

I doubt if the description of this disease, as given by Eberle in his work on Practice, has been surpassed by any modern author. By the way, I will venture the opinion that this work—more than 50 years old—in so far as its description of the symptoms of disease is concerned, has never been equalled by any subsequent writer.

It is highly probable that the remote seat of the affection exists in the medulla oblongata, and radiates from this point through the pneumogastric nerve to its pulmonary branches, influencing or producing the contraction of the minute bronchial tubes and air cells.

This nerve, gentlemen, sometimes called the 8th or par vagum, you have been taught, presides over the function of respiration. It passes off from the medulla oblongata; it emerges from the cranium through the foramen lacerum; it passes down the side of the neck, enters the chest and distributes branches to the pharynx, the larynx, the esophagus, the lungs and the stomach. From these extensive ramifications you will readily infer the important and varied functions of this nerve and its relations to the respiratory organs.

A recent writer of eminence in the profession has remarked: "That the essential factor in asthma seems clearly to be a condition of morbid sensibility or of actual disease of the nervous filaments supplying the ramifications of the bronchial tubes, and perhaps of the thoracic ganglia of the sympathetic. If such a state of morbid irritability should exist, however induced, it would be easy to understand how a paroxysm of spasmodic dyspnœa might be produced either by reflex irritation (as from the stomach), or from direct irritation—as from sudden congestion or catarrhal inflammation of the bronchial mucous membrane."

I would remark that this theory very plausibly explains how that slight impressions from without, as a damp or unfavorable atmosphere, dust, pollen, and odors of different kinds might excite the excessively sensitive bronchial nerves of the asthmatic, and how it is that a change to a different atmosphere, or a removal of the various irritants mentioned, might be followed by the relief of the patient.

The morbid sensibility of the bronchial nerves to which is ascribed the attacks, is not unfrequently inherited, passing from the parents or the remote ancestry to the child. It is perhaps more frequently acquired, resulting from any cause that may induce a morbid condition of the bronchial nerves.

Whatever may be the cause of the disease, it is more common in men than in women. It very frequently commences with some irregularity of diet, giving rise to indigestion and unfavorably impressing the branches of the pneumogastric nerve distributed to the stomach.

While in other subjects a like impression might be followed by a colic, a headache, an intercostal neuralgia or other disorder, in the unfortunate class of individuals of whom we are speaking, it occasions an attack of asthma, When an attack has once occurred, it is very apt to be repeated until finally an asthmatic habit is established and becomes confirmed. The habit once established, very slight causes may bring on an attack. I may mention as among the most frequent causes: mental emotion, change of place, violent exertion, suppressed discharges, catarrh, etc.

Some have attributed the disease to a paralytic condition of the pneumogastric nerve. The fact that a division of this nerve gives rise to extreme dyspnœa is the argument made in support of this theory.

But dyspnœa does not, in itself, constitute asthma, and may exist in heart disease, in emphysema, or from mechanical obstructions of any kind to the free action of the lungs; while in idiopathic asthma these conditions do not necessarily exist.

Asthma is frequenely attributed to organic heart disease, and that asthmatic symptoms may result from these causes, there can be little doubt. Yet in the large majority of instances the heart complication is the result and not the cause of the affection. That the impediment to the free entrance of the blood into the lungs caused by the constriction of the air cells, and the consequent regurgitation or damming up of the blood in the right cavities of the heart, should, after a long continuance of the malady, produce enlargement or valvular difficulty,is not surprising, and indeed it will almost unavoidably occur. So, that there are perhaps very few confirmed asthmatics who are free from heart complication in some degree.

I may mention other conditions as resulting from the disease. The one is the barrel-shaped chest; the frequent and violent distention of the lungs having the effect of permanently enlarging the shape of the thoracic walls, which are full, large and round, giving a drum-like sound on percussion. A peculiar glaring or popped expression of the eye is also a mark of the asthmatic.

*Emphysema.*—Another result of asthma is that of *emphysema.* This word is from the Greek *emphusao*, to inflate. In this affection the air cells are often permanently dilated, giving rise to what is called vesicular emphysema, and sometimes the cells are ruptured and coalesce, giving rise to infiltration of air into the areolar or interlobular tissue. This last condition is termed interlobular emphysema. Emphysema, though often a resultant of asthma, is treated in our authorities as a distinct affection. It may be distinguished from asthma by the fact that the dyspnœa is constant and continuous, while in uncomplicated asthma it is occasional or paroxysmal.

If asthma is complicated with emphysema, it is usually regarded as incurable, otherwise the prognosis is not necessarily hopeless. It is admitted, however, that asthma, though rarely fatal, is seldom permanently cured.

*Hay Fever.*—There is a form of catarrh or bronchitis to which the name of hay fever or hay asthma has been given. It is sometimes called summer catarrh, because it commonly makes its appearance in the summer season; sometimes in the early fall, not unfrequently recurring on the same day of the month with each successive attack. Though often classed and treated as asthma, it is more properly a periodic catarrhal affection, attended by sneezing, running at the nose, sniffling, more or less bronchial irritation and cough. As it usually comes on about the hay season, it was supposed to be caused by the odor or dust from hay, hence its name. The pollen of the rag-weed is said also to cause the disease. Another and not an improbable theory is that it proceeds from atmospheric germs or organisms. Temporary relief may be obtained by snuffing morphine into the nostrils, but the best treatment known is probably the use of quinine, both internally and as a nasal lotion. This and the removal of the patient to an elevated region and pure atmosphere, has been found most efficient.

Prof. Helmholtz, as far back as 1868, advanced the theory of low organisms as the cause of hay fever. He discovered in his own case, certain vibrio-like bodies with the immersion lens of a very good Hartnack's microscope. He found that a neutral solution of the sulphate of quinine locally applied gave prompt relief. He lay flat on his back, keeping his head low, and poured, with a pipette, about four cubic centimetres into both nostrils, turning his head about to let the liquid flow in all directions. This treatment repeated three times a day for a week relieved him for the entire season.

Chlorate of potash in solution with morphine as a nasal douche is highly recommended.

Dr. Weber, of Philadelphia, recommends the following formula as very efficient in hay fever, and I judge from the combination that it would prove useful also in asthma—

| | | |
|---|---|---|
| ℞ | Ext. hyoscyami | gr. xii, |
| | Potass. iodid | ʒj, |
| | Potass. bicarb. | ʒij, |
| | Ext. glycyrrhizæ | ʒiv, |
| | Aquæ anisi | ℥ivss. |

M. S. A desertspoonful every four hours during the day and night until relieved. May be continued a week.

It is usual to divide the treatment of asthma into: first, that which is proper during the paroxysm; and, second, that which should be adopted during the intervals.

The remedies recommended for the relief of the paroxysm are legion. I will mention those principally relied upon, and will give you such of the recent formulæ as seem most valuable, and which, by their variety, may be found applicable to the different phases of the disease resulting from constitutional peculiarities, or from the differences of existing complications.

*Lobelia.*—This drug has long been regarded as the best remedy in asthma. The dose is one teaspoonful of the tincture every half hour until relief follows. If vomiting results without relief, it is usually abandoned and another agent tried.

A good formula for administering lobelia is the following—

| | | |
|---|---|---|
| ℞ | Tinct. lobelia, | aa ℥j. |
| | Tinct. hyoscyami, | |
| | Spiritus etheris compositi, | |
| | Syrupi tolutani, | |

M. A teaspoonful every half hour until some impression is made, and then every one to two hours. This remedy should be associated with sinapisms to the upper half of the spine and to the chest.

Another formula suggested is the following, and will suit a large number of cases, especially when occurring in children and hysterical females—

| | | |
|---|---|---|
| ℞ | Tinct. lobeliæ, | aa ℥j. |
| | Tinct. assafœtidæ, | |
| | Tinct. valerianæ, | |

Dose for an adult one tablespoonful every one to two hours until some relief follows, then not so often.

A valuable formula for asthma is the following—

| | | |
|---|---|---|
| ℞ | Iodid potass | ʒij, |
| | Sulphate of morphia, | gr. j, |
| | Comp. syr. squills, | aa ℥ij. |
| | Tinct. lobelia | |

M. Dose, one teaspoonful every two to three hours.

Hypodermic morphia has, of late, been found very satisfactory in giving prompt relief to the paroxysm—

℞ Muriate morph,... gr. ¼,

Water, ... q. s.

Use hypodermically.

℞ Potassii nitras, ... }

Pulv. anisi fructus, ... } aa ℥ss,

Pulv. stramonii fol, ... ℥j.

Misce. A thimbleful of the powder placed on a plate is pinched into a conical shape and lighted at the top. It burns like a pastile, and is held near the patient, who inhales the fumes. The smoking of the leaves of stramonium often gives relief.

The iodide of potash in large doses, combined with the tincture of gelsemium and belladonna, has been found a very efficient remedy for the relief of the paroxysm in obstinate cases—

℞ Iodide potass ... gr. x to xx,
Tinct. belladonna ... gtt. v,
Tinct. gelsemium,... gtt. xv.

In half a glass of water.

This at a single dose is usually sufficient, but it may be repeated once or twice in violent cases, if necessary.

The inhalation of chloroform, though it often fails, occasionally gives prompt relief to the paroxysm.

For treatment during the intervals of the paroxysms, it is advised to change, if possible, the location of the patient to a different atmosphere. It being found in many cases that the paroxysms are thus prevented, the relief being permanent, or continuing so long at least as the patient remains in an atmosphere adapted to his condition.

The remedies found most useful in warding off the paroxysms are: first, the sulphate of quinine, which is particularly adapted to cases in which malarial complication is present; and to cases wherein the intervals are brief between the attacks. It is best used in three to five grain doses three times per day in solution with, or used in conjunction with, three to five drops of nitro-muriatic acid.

Another excellent method of using the quinine is in the following combination—

℞ Sulph. quinine., ... }

Pyrophosphate iron, ... } aa gr. 60,

Strychnia,... gr. j.

M. Make pills No. 50.

Take one three times per day. This is specially adapted to cases complicated with indigestion or constipation.

Another useful agent in the interval is the oxide of zinc in one grain doses three times per day. The valerianate of zinc is also a good remedy.

Fowler's solution of arsenic, two to five drops three times a day until slight constitutional impression is perceived then suspended and used again, has been successful in some cases.

These are the sheet-anchors in asthma, and may be varied or combined with other agents according to circumstances. Often the best remedies will fail if existing complications are disregarded. The secretions must be looked after and regulated by appropriate means. Disorders of the liver and stomach may require attention; and in many cases success will be greatly promoted by the use of mercurials and purgatives as preliminary means. The blue pill may be given, or the bichloride of mercury in minute doses, for a time, will often be required to put the patient in a good conditiou for the use of other means, whether they are designed for the immediate suppression of the paroxysm or as prophylactic to their recurrence.

If the patient be of a plethoric habit, the tonics may not be needed, but rather a restriction of diet, or the use of saline purgatives will be better. In former times the lancet was used with advantage in such cases, and if the case be urgent or extreme, we should not hesitate to take blood from a patient of this character.

An important and not unfrequent complication is a torpid skin, giving rise to suppressed or imperfect perspiration, and thus tending to congestion or functional disturbance of internal organs affecting the lungs and keeping up the attacks. This should be met by warm or cold baths, friction and methods calculated to restore the function of the skin.

*Vesicular Emphysema*—This condition is treated by the tonic and alterative agents which I have mentioned as appropriate to asthma. It is rarely permanently relieved. The young sometimes seem to be cured or to out-grow it. In the case of the little girl I have shown you, I commenced the treatment with a small dose of calomel and podophylin given in syrup of rhubarb. This was several times repeated, at intervals of about twice a week, until the secretions were in better condition. The relief from this prescription was very considerable—I should mention that an assafœtida pill was given at night as an antispasmodic and to procure rest. I then put the patient on the following prescription, under which she steadily improved. As there is in this case no decided heart lesion and the patient is young, it is believed that she is permanently cured. The remedy given was the following—

℞ Com. syr. squills,.............................. ℥j,

Brom. potass................................. ʒj,

Com. tict. cinchona,.......................} aa ℥ij,

Fluid ext. valerian,.........................}

Tinct. digitalis,...... ............................ ʒij,

M. Take a teaspoonful three times a day.

There are many new remedies which have been presented through the journals for asthma. One of these is the *Quebracho Blancho*, which is a tree found in the Argentine Republic, and is now being introduced into this country. It is used by the natives as a remedy for asthma, and some tests which have been made with it in our country indicate that it possesses great power over the respiration and circulation, and it is believed to be well adapted for the relief of almost every form of dyspnœa.

The semi-solid oil that accumulates in the tubing of the oil wells, in doses of five grains, made into pills with pulverized gentian and taken three times a day, is reported as having permanently cured a case in Oregon.

Painting the sides of the neck with iodine over the course of the pneumogastric nerves has been found to give prompt relief to the paroxysm.

The inhalation of the iodide of ethyl is reported in a French journal, as wonderfully efficacious in relieving the paroxysms of asthma. Only 6 to 10 drops are to be inspired by the patient, and this may be repeated several times a day.

*Grindelia Robusta*, a new remedy introduced from the Pacific coast, has been recently highly recommended for asthma. Dose of the tincture from a half to one drachm.

